# Rifabutin loaded inhalable β-glucan microparticle based drug delivery system for pulmonary TB

**DOI:** 10.1038/s41598-024-66634-5

**Published:** 2024-07-16

**Authors:** Firoz Ahmad, Shad Ahmad, Tarun Kumar Upadhyay, Sanjay Singh, Mohd Khubaib, Jyotsna Singh, Mohd Saeed, Irfan Ahmad, Lamya Ahmed Al-Keridis, Rolee Sharma

**Affiliations:** 1 IIRC-3 Immunobiochemistry Lab, Department of Biosciences, Integral University, Lucknow, UP 226026 India; 2 Department of Clinical Immunology & Rheumatology, Sanjay Gandhi Post Graduate Institute of Medical Sciences, Lucknow, UP 226014 India; 3 Department of Biochemistry, Dr. Ram Manohar Lohia Avadh University, Faizabad, UP 224001 India; 4 Department of Life Sciences, Parul Institute of Applied Sciences & Research and Development Cell, Parul University, Vadodara, Gujarat 391760 India; 5 Pharmaceutics and Pharmacokinetics Division, CSIR-CDRI, Lucknow, UP 226201 India; 6 Inhalation Toxicology Facility, CSIR-Indian Institute of Toxicology Research, Lucknow, UP 226008 India; 7 Department of Biology, College of Sciences, University of Hail, 34464 Hail, Saudi Arabia; 8 Department of Clinical Laboratory Sciences, College of Applied Medical Science, King Khalid University, Abha, Saudi Arabia; 9 Department of Biology, Faculty of Science, Princess Nourah bint Abdulrahman University, P.O.Box 84428, 11671 Riyadh, Saudi Arabia; 10 Department of Life Sciences and Biotechnology, CSJM University, Kanpur, UP 228024 India

**Keywords:** Anti- TB drug delivery, Alveolar macrophages, Rifabutin, β-Glucan particles, Biochemistry, Drug discovery, Immunology, Microbiology, Diseases, Medical research, Materials science, Nanoscience and technology

## Abstract

Inhalable microparticle-based anti TB drug delivery systems are being investigated extensively for Tuberculosis [TB] treatment as they offer efficient and deep lung deposition with several advantages over conventional routes. It can reduce the drug dose, treatment duration and toxic effects and optimize the drug bioavailability. Yeast derived β-glucan is a β-[1–3/1–6] linked biocompatible polymer and used as carrier for various biomolecules. Due to presence of glucan chains, particulate glucans act as PAMP and thereby gets internalized via receptor mediated phagocytosis by the macrophages. In this study, β-glucan microparticles were prepared by adding l-leucine as excipient, and exhibited 70% drug [Rifabutin] loading efficiency. Further, the sizing and SEM data of particles revealed a size of 2–4 µm with spherical dimensions. The FTIR and HPLC data confirmed the β-glucan composition and drug encapsulations efficiency of the particles. The mass median aerodynamic diameter [MMAD] and geometric standard deviation [GSD] data indicated that these particles are inhalable in nature and have better thermal stability as per DSC thermogram. These particles were found to be non-toxic upto a concentration of 80 µg/ml and were found to be readily phagocytosed by human macrophage cells in-vitro as well as in-vivo by lung alveolar macrophage. This study provides a framework for future design of inhalable β-glucan particle based host-directed drug delivery system against pulmonary TB.

## Purpose, rationale and limitations

The purpose of this study was to develop a novel drug delivery system of anti-TB drug, Rifabutin, in the form of Inhalable β-glucan micro particles with a view to achieve improved treatment of pulmonary tuberculosis (TB).Specifically, the study aims to investigate the feasibility and efficacy of utilizing inhalable β-glucan microparticles as carriers for rifabutin, a potent antibiotic used in TB therapy. The ultimate goal is to enhance the therapeutic outcomes of rifabutin by improving its pulmonary delivery, thereby addressing the challenges associated with conventional oral administration, such as poor bioavailability and systemic side effects. The rationale for this study is grounded in the need for innovative approaches to improve the treatment of pulmonary TB, a global health concern. Rifabutin is a first-line of antibiotic use for the TB treatment, but its efficacy is hindered by issues such as poor solubility and low bioavailability. By encapsulating rifabutin within inhalable β-glucan micro particles, the study seeks to overcome these challenges and achieve targeted drug delivery to the lungs, the primary site of infection in pulmonary TB. The rationale for utilizing β-glucans lies in their biocompatibility, immunomodulatory properties, and ability to serve as effective carriers for pulmonary drug delivery. Through this approach, the study aims to enhance the therapeutic efficacy of rifabutin while minimizing systemic side effects and improving patient compliance. One limitation of the study may be the optimization of the particle size of β-glucan micro particles for efficient lung deposition. Achieving the ideal particle size distribution to ensure deep lung penetration and uniform drug delivery may require extensive formulation optimization and characterization. While β-glucans are generally considered non-toxic and biocompatible, potential immunogenicity or adverse reactions associated with their inhalation route of administration must be carefully evaluated. Comprehensive preclinical studies are necessary to assess the safety profile of the inhalable β-glucan micro particle system to transitioning from preclinical studies to clinical trials. Despite of these limitations, the study represents a significant step towards addressing the unmet clinical needs in TB therapy by leveraging the advantages of inhalable β-glucan micro particles as carriers for rifabutin pulmonary delivery. Further research and development efforts are warranted to optimize formulation parameters, assess safety and efficacy in preclinical models, and advance towards clinical translation.

### Introduction

Tuberculosis [TB] still persists as one of the most common infectious diseases in the world, causing 10 million deaths worldwide annually^1^. despite of the clinical advancement in medical sciences, TB exists as an alarming threat for mankind due to the emergence of multidrug resistance [MDR-TB]/ extensive drug resistance [XDR-TB] and total drug resistance [TDR-TB], further increasing the global bio burden of this fatal disease and worsening the situation^2–4^. The current conventional oral therapy is inefficient as it often fails to completely cure patients, due to multiple reasons such as poor drug solubility, less drug bioavailability to the site of infection, drug degradation as well as toxicity and prolonged treatment duration, leading to non-compliance issues. In this context, recent advancements in the form of biocompatible and biodegradable nano- or micro-particle [NIM] based targeted drug delivery systems forms a promising approach for the mitigation of these issues^5,6^. β-glucans are one of the most abundant polysaccharides found in the microbial cell walls of bacteria, fungi, cereals, etc., and it consist of long glucose chains with β-[1–3, 1–6] glycosidic linkages. These have been granted the Generally Regarded As Safe [GRAS] status by the USFDA and have also been recommended by European Food Safety Authority [EFSA] for boosting the immune function^7^. β-glucans are also consider as biological response modifier [BRM] to modulates immune system by promoting the maturation of DC cells, cytokine secretion, and regulating adaptive immune responses^8,9^ Recently, the yeast derived glucan microparticles have gained attention as biodegradable and biocompatible drug delivery systems that are porous in nature and thus allow encapsulation of various molecules including anti-TB drug within these particles^10–12^. These microparticles also serve as pathogen associated molecular patterns [PAMPs], and are recognized by the macrophage receptors including Dectin-1, CR-3 and FC-γ^13^. They are thereby efficiently uptaken by macrophage cells and further assist in the release of the encapsulated drug in targeted manner within the macrophages^14^.

Pulmonary drug delivery in form of inhalable particles offers several advantages such as improved drug bioavailability, enhanced patient compatibility, reduced systemic side effects and prolonged drug action over conventional oral therapy^15–17^. The size of the particles plays an important role in deep lung delivery, as well as in their amenability to uptake by resident alveolar macrophages, leading to intracellular release of the encapsulated drug molecules^18,19^. Studies reveal that for efficient pulmonary drug delivery and deposition during inhalation, the mass median aerodynamic diameter [MMAD] of particles should be less than 5 µm, with geometric standard deviation [GSD] values between 0.5 and 2.5 µm^20–22^.

Spray drying is generally used for the preparation of the inhalable microparticles; it consists of the three steps: atomization, drying, and collection^23–25^. L-leucine addition during particle preparation is reported to improve the dispersibility of particles during spray drying and the aerosolization and physical stability of microparticles, due to its enrichment on particle surfaces and intermolecular interactions with drugs^26–28^. The objective of this study was to develop β-glucan particles loaded with rifabutin and assess their inhalable characteristics using both in-vivo and in-vitro method for the betterment of pulmonary TB management. The development of this Rifabutin-loaded Inhalable β-glucan micro particle-based drug delivery system represents a significant advancement in TB therapy, offering a targeted and efficient approach to combatting this infectious disease. Through further research and clinical trials, this innovative system holds the potential to revolutionize the treatment of pulmonary TB, ultimately improving patient outcomes and reducing the global burden of this deadly disease^29,30^.

## Results

### Microparticle size distribution and morphology

**Figure 1:**
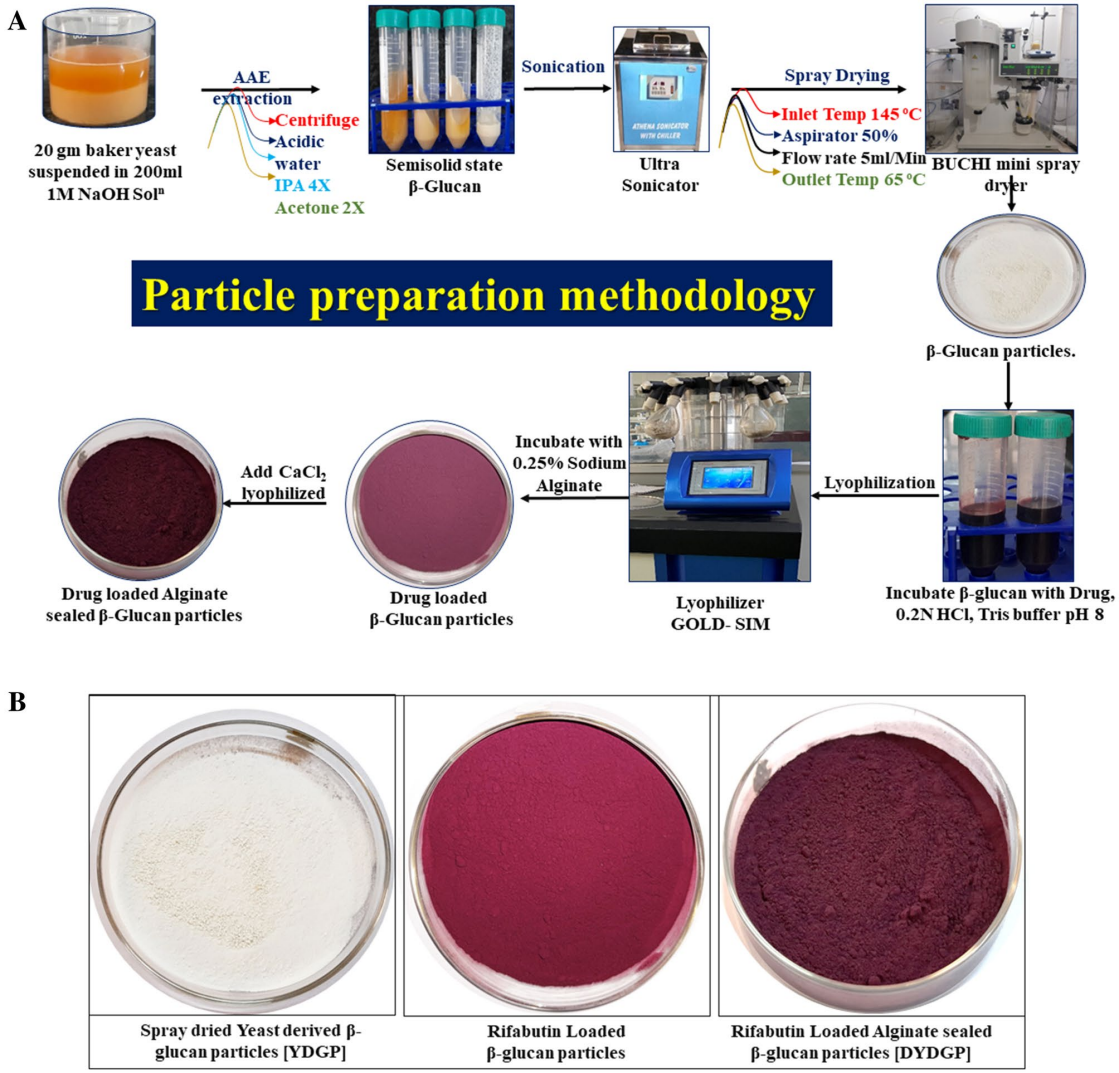
(**A**): Inhalable Microparticle preparation methodology, (extraction /drug loading/Alginate sealed). (**B**): Representative images of Blank, drug loaded and sealed microparticle.

**Figure 2:**
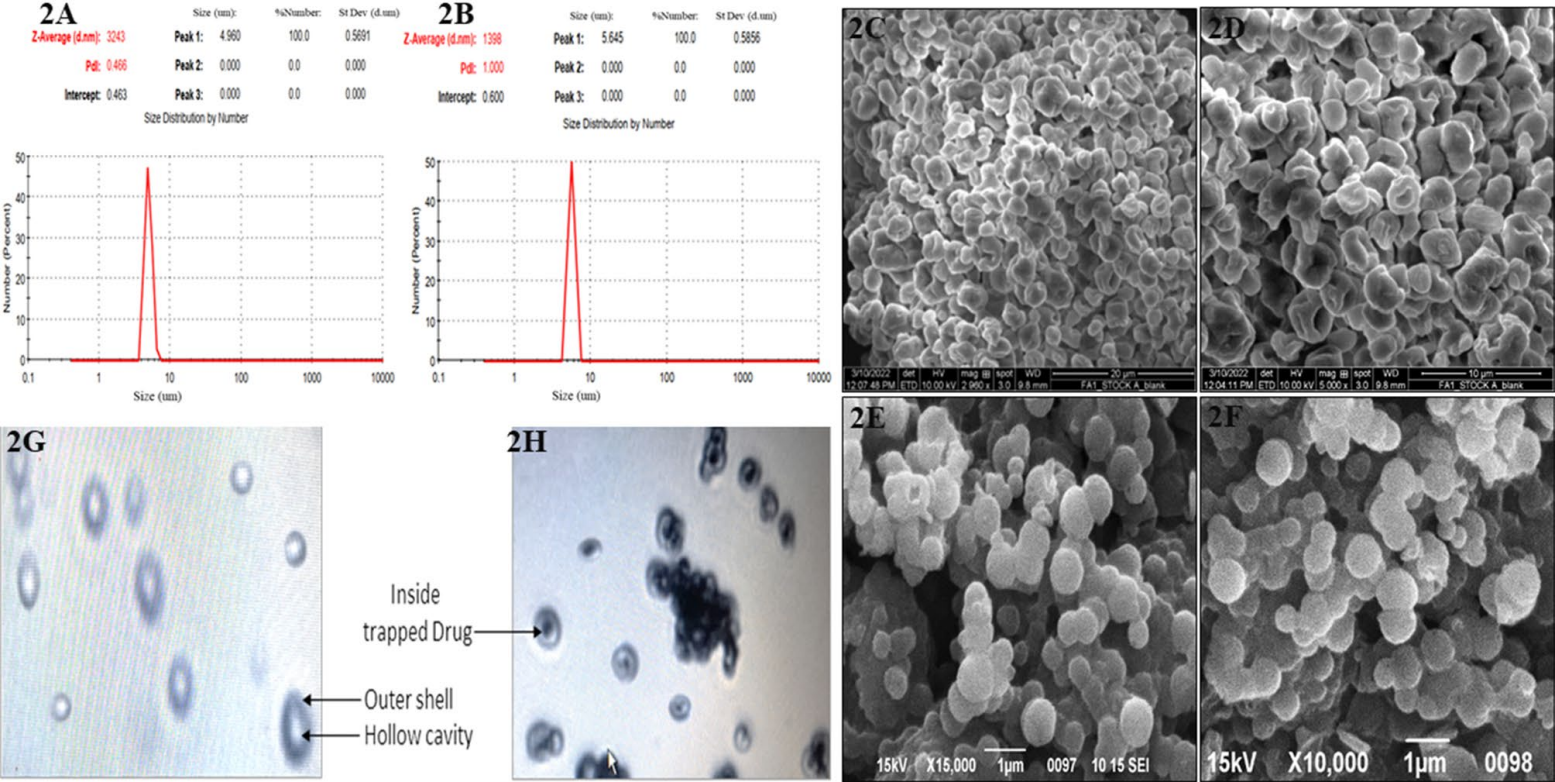
Size distribution of the particle done by Malvern Mastersizer. (**A**) Blank β- glucan, (**B**) Drug loaded Inhalable microparticles. SEM Images Scanning Electron Micrograph of Blank β- glucan (**C**, **D**) and drug loaded (**E**, **F**) particles at different magnifications. Microscopic examination microparticles, (**G**) Blank β- glucan, (**H**) Drug loaded particles.

The extraction method of glucan microparticle from baker yeast has shown in the [Fig. 1A]. The extracted blank glucan microparticle [YDGP] and drug loaded [DYDGP], has shown in [Fig. 1B]. Blank YDGP has shown as off white in color while intermediated and drug loaded [DYDGP] glucan microparticle shown as light pink red to dark pink red in color. The average particle size distribution for both [YDGP] and [DYDGP], was found to be in the range between 1 and 5 µm. The drug loaded particles were found to have unimodal particle distribution [poly dispersed], with a higher fraction [88%] of particles in the 1–5 µm range, and a very small fraction of the particles [5–10%] was found above the 5 µm [Fig. 2A,B]. The percentage value of the [DYDGP] microparticles represents the 10% of particles in the powders are smaller than this size d(0.1), 50% of the total particles are smaller than this size d(0.5) and 90% of the total particles are smaller than this size d(0.9) are slightly different for the DYDGP batch A it is [0.210 µm, 5.800 µm, 19.315 µm] while DYDGP batch B [0.908 µm, 1.413 µm, 3.496 µm] respectively. The size and surface morphology of these particles were also examined by SEM, which revealed that while the blank particles were collapsed and shriveled due to hollow and porous nature, with an uneven surface, while the drug loaded particles were spherical in nature due to entrapped drug within pores makes the particles to expand the overall particle. Due to the agglomeration some particle are fused it may be due the calcium-alginate sealing but the overall particles are spherical in nature [Fig. 2C,D,E,F]. For the further examination of the microparticle the has shown the hollow cavity within blank particles and drug encapsulation within these particles was also visualized under a bright field microscope [Fig. 2G,H].

### FT-IR spectra of particles

**Figure 3:**
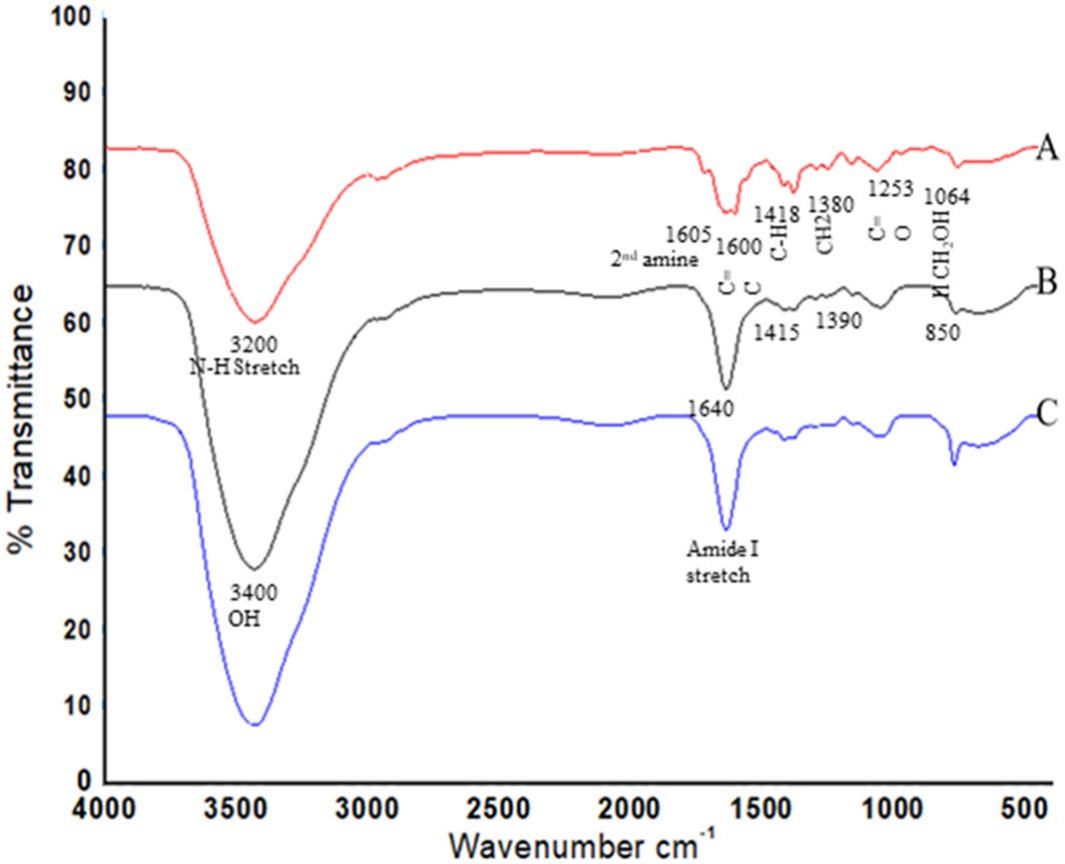
FTIR spectra of extracted (**A**) Rifabutin, (**B**) β- glucan particles, (**C**) Drug loaded Alginate sealed particles.

The FT-IR spectra of these particles confirmed the presence of β-glucan and Rifabutin specific functional groups within the prepared formulations Regarding the glucan specific functional groups, the absorbance peak at 928–1200 cm^−1^, due to C–C and C=O stretching vibrations confirmed the presence of pyranoids rings, indicating polysaccharide nature of the particles. The absorption peak at 3400.06 cm^−1^ represents the hydroxyl group of OH stretch, while the signals at 1215.72 cm^–1^ indicate C–H stretch and CH_2_OH stretch. The absorption peak at 850.84 cm^−1^ is characteristic of β-glycosidic bonds, i.e. [C_1_–H] deformation mode, and, thereby, indicates the presence of β-glucans. Absorption peak at 760.71 cm^−1^ shows presence of α-glycosidic bond. The peaks 1628 cm^−1^ and 1406 cm^−1^ indicate presence of a small fraction of mannosylated residual proteins^31,32^. The FTIR spectra of Rifabutin-loaded glucan particles revealed Rifabutin specific functional groups with peaks at 1064.67 cm^−1^ indicating the presence of secondary alcohol and, peaks at 1253.91 cm^−1^ for the carbonyl stretch of the C=O groups, at 1718.88 cm^−1^, 1638.67 cm^−1^ vibration for the C=O stretching. Absorbance peaks at 3200.36 cm^−1^, 1605.24 cm^−1^ represent the N–H stretch and N–H deformation 2nd amine. Other peaks at 1418.84 cm^−1^, 1380.95 cm^−1^ indicate the C–H deformation of CH_2 ­_group of rifabutin and at 1600.24 cm^−1^ for vibration of C=C stretch^33^. The FTIR spectra of drug loaded alginate sealed particles exhibit peaks that overlap with the peaks of glucan and rifabutin specific groups, thereby confirming no change in the chemical structure of rifabutin, and indicating no loss in biophysical property of Rifabutin during its encapsulation within glucan particles [Fig. 3].

### Differential scanning calorimetry [DSC] analysis

**Figure 4:**
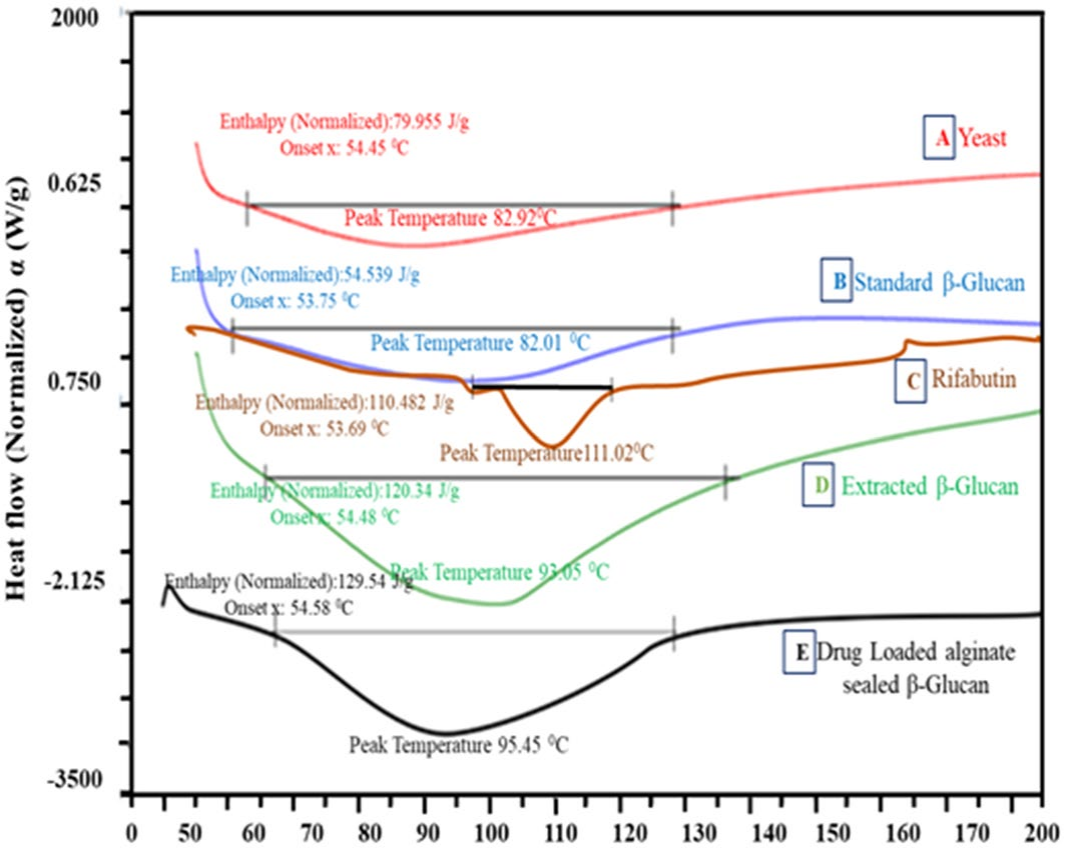
Differential scanning calorimetry (DSC) Curve of particles. (**A**) Baker Yeast, (**B**) Standard β- glucan, (**C**) Rifabutin, (**D**) Extracted β- glucan, (**E**) Drug loaded microparticles.

Differential Scanning Calorimetry was used to analyze the purity and thermal stability analysis of the particles that showed broad endothermic peaks from 50 to 128 °C corresponding to the breaking of molecular hydrogen bond and evaporation of residual water molecules from β-glucans. Drug loaded and sealed particles were found to have higher glass transition temperature in comparison to the standard and extracted glucan [Fig. 4].

### Particle drug content

**Figure 5:**
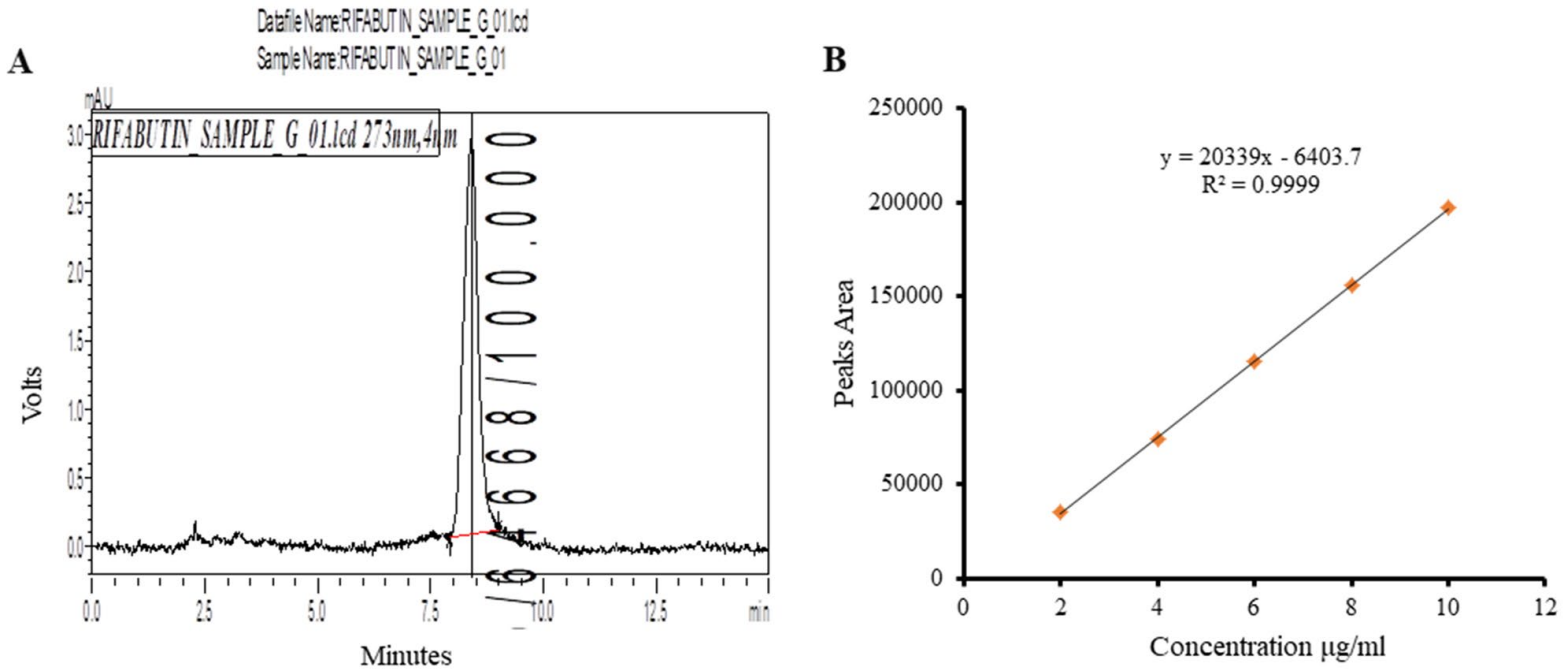
Quantification of the drug By HPLC. (**A**) HPLC graph (**B**) Calibration curve.

Drug content estimation by HPLC revealed a loading efficiency of 68–70% within [DYDGP] formulations [Fig. 5A,B] the drug loading efficiency of the formulation was seen to be greatly improved due to addition of leucine it’s enhanced the flow property of particle and during drug loading the surface area of blank β-glucan particle exposed with drug so thatswhy [%] of drug loading efficiency increased. [Table 1].

**Table 1:** Spray drying Parameter & Characteristics of Blank (YDGP) β-glucan particle.

Blank	Inlet Temp (°C)	Aspirator (%)	Outlet Temp (°C)	Pump Flow rate (ml/min)	Yield in (gram)	Yield in (%)	Polymer Drug ratio (gram)	Leucine conc. in (mg)/gram
YDGP-A	145	50	45	5	3.48	17.5	1:1	100
YDGP-B	150	50	50	5	4.08	20.5	1:1	200
YDGP-C	155	50	55	5	4.48	22.5	1:1	300

Drug	d0.1 (µm)	d0.5 (µm)	d0.9 (µm)	Drug loading efficiency (%)	MMAD (μm)	GSD (μm)		
Characteristics of Rifabutin drug loaded (DYDGP) β-glucan particle								
DYDGP-A	0.210	5.800	19.315	67	1.58	1.43		
DYDGP-B	0.374	4.320	6.341	68	2.07	1.81		
DYDGP-C	0.908	1.413	3.496	70	1.02	1.63		

### Aerodynamic behavior of glucan particle

**Figure 6:**
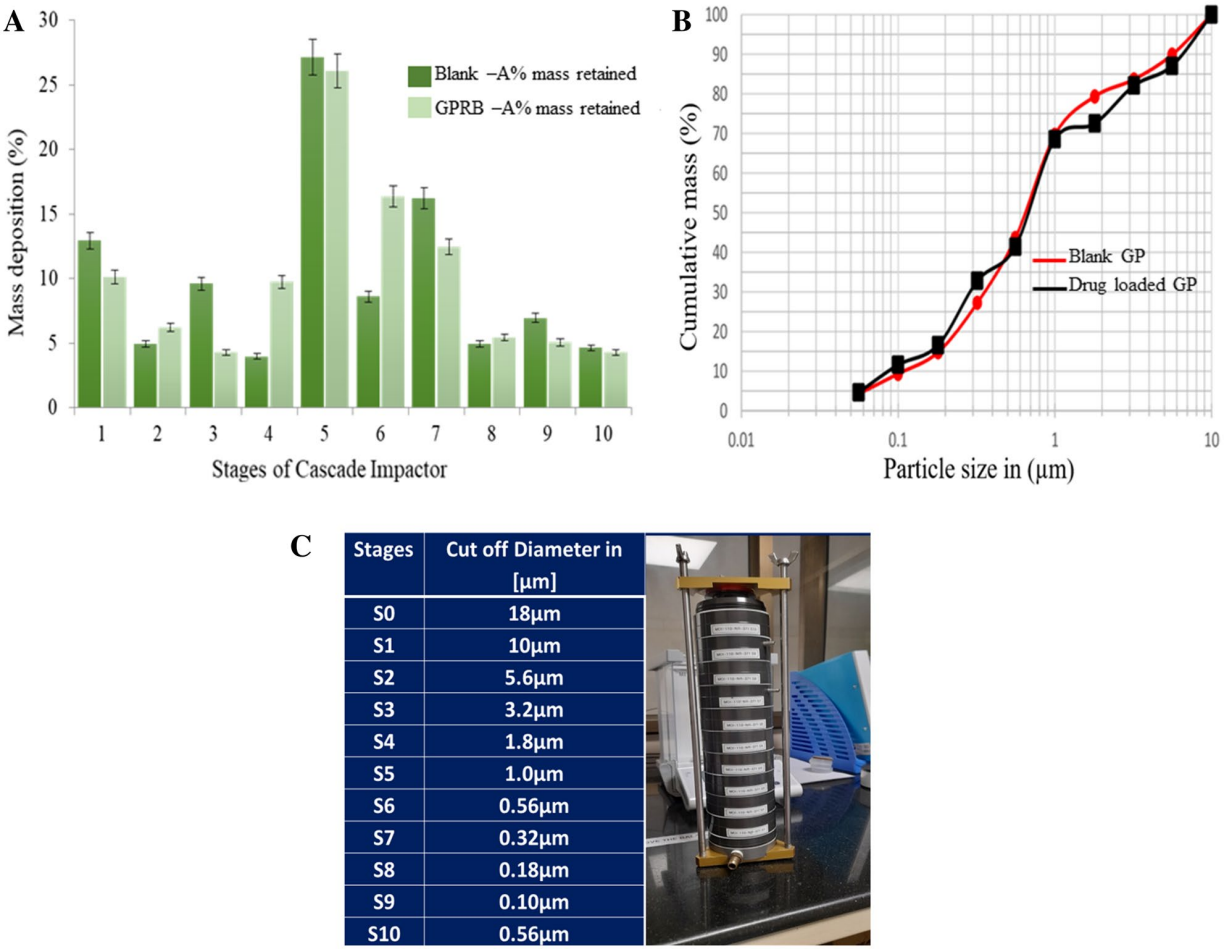
Deposition profile of Drug loaded Inhalable microparticles distribution in each stage ACI Impactor (**A**), Percentage of microparticle deposition, (**B**) Cumulative size and mass of blank and drug loaded glucan particles, (**C**) Cut off diameter of impactor.

The mass median aerodynamic diameter [MMAD], and GSD value of the blank and drug loaded particles was found to be 1.01–1.62 μm and 1.02–2.07 μm respectively [Table 1]. The mass median aerodynamic diameter [MMAD] and geometric standard deviation [GSD] of the particles were calculated by sigmoid curve fitting of the values of cumulative mass percent of particles undersize deposited on the stages against the log^10^ values of effective cut-off diameter of the each stages cascade impactor is showing in. [Fig. 6A,B,C].

### In- vitro and in- vivo phagocytic uptake

**Figure 7:**
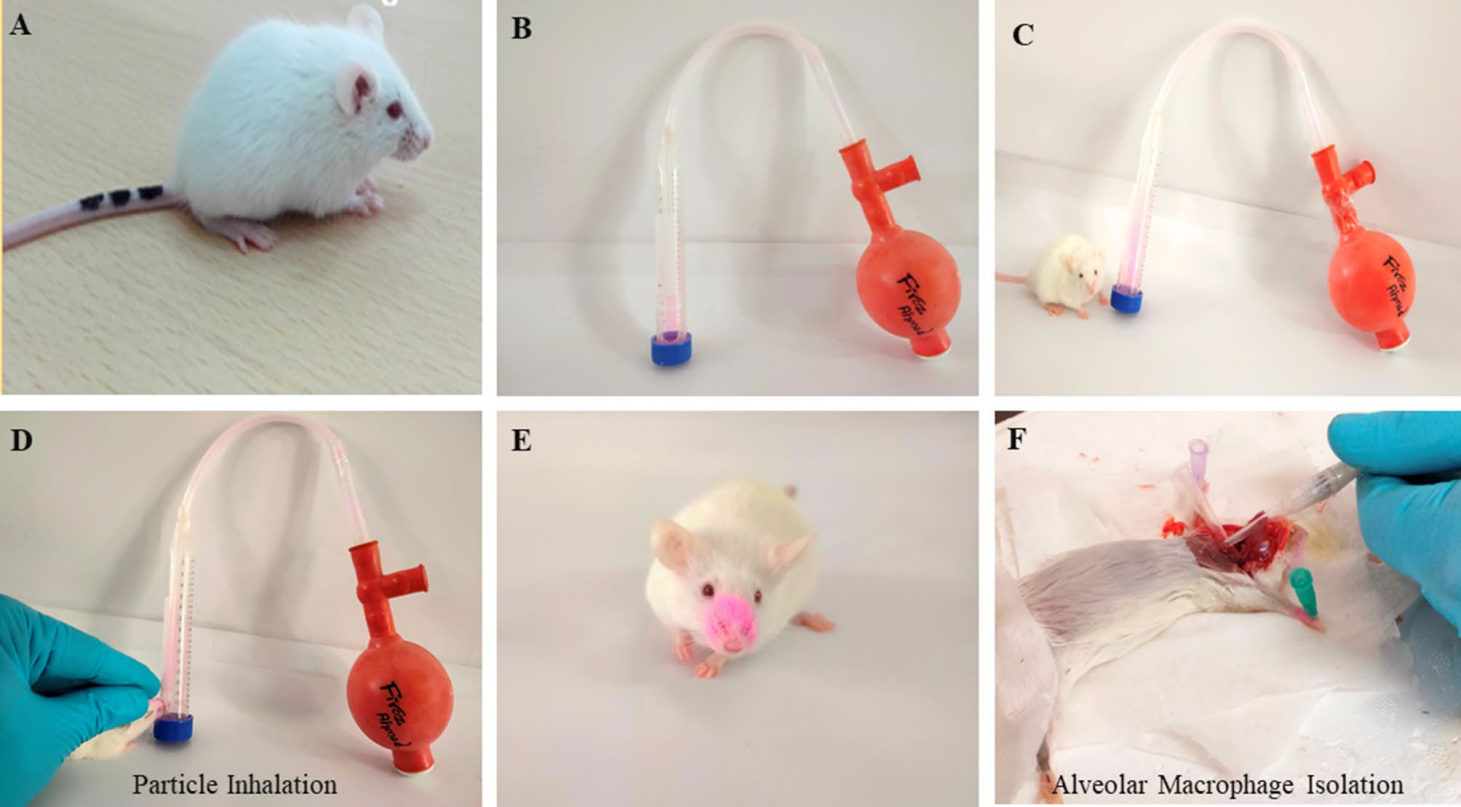
Inhalation of particle & isolation of lung alveolar macrophage cell from Bronchoalveolar Fluid.

**Figure 8:**
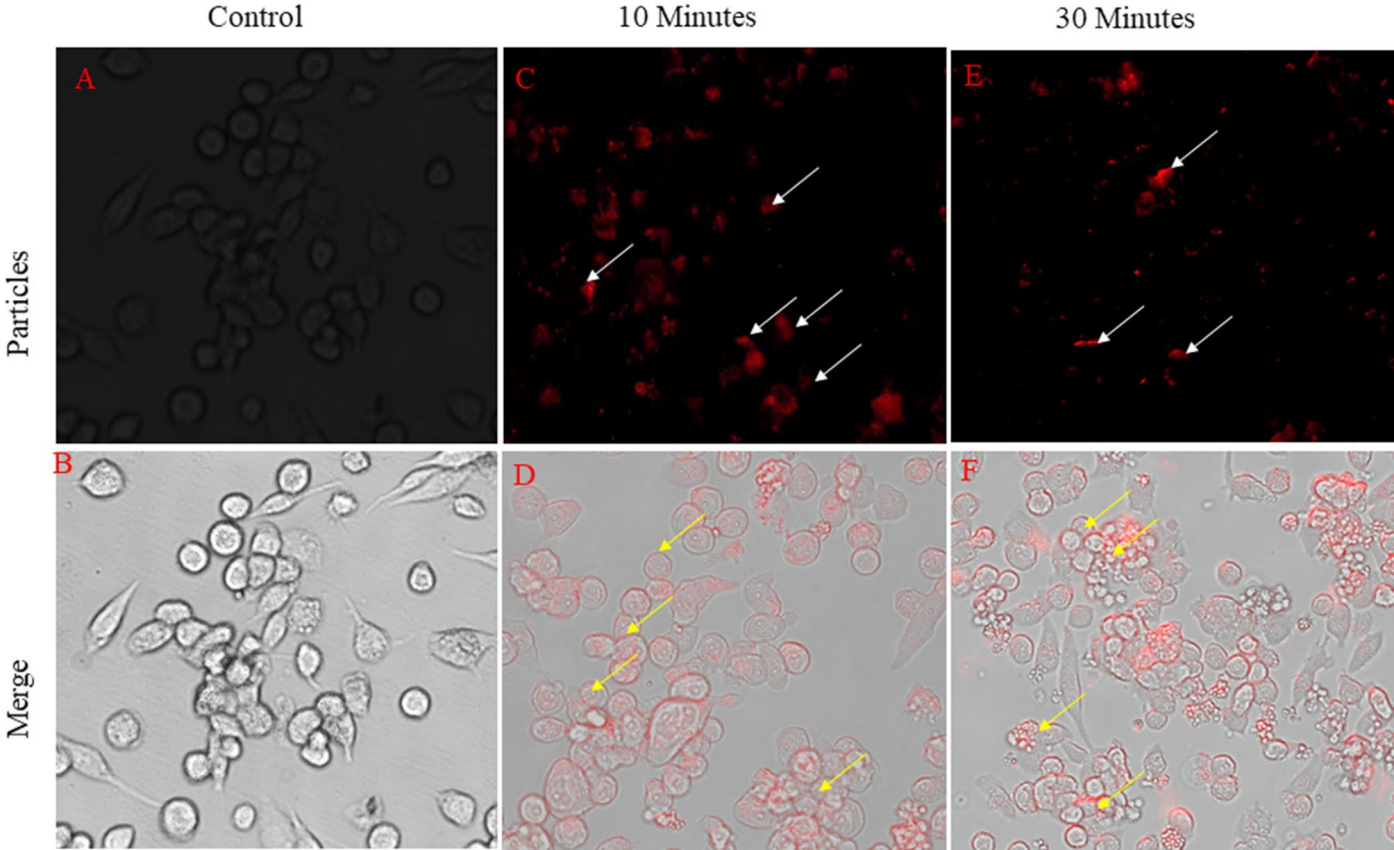
Evaluation of in-vitro Phagocytic uptake of the Rhodamine labelled Drug loaded Inhalable microparticles within THP-1 cells. (**A**) Control cell, (**B**) 10 Minutes, (**C**) 30 Minutes.

**Figure 9:**
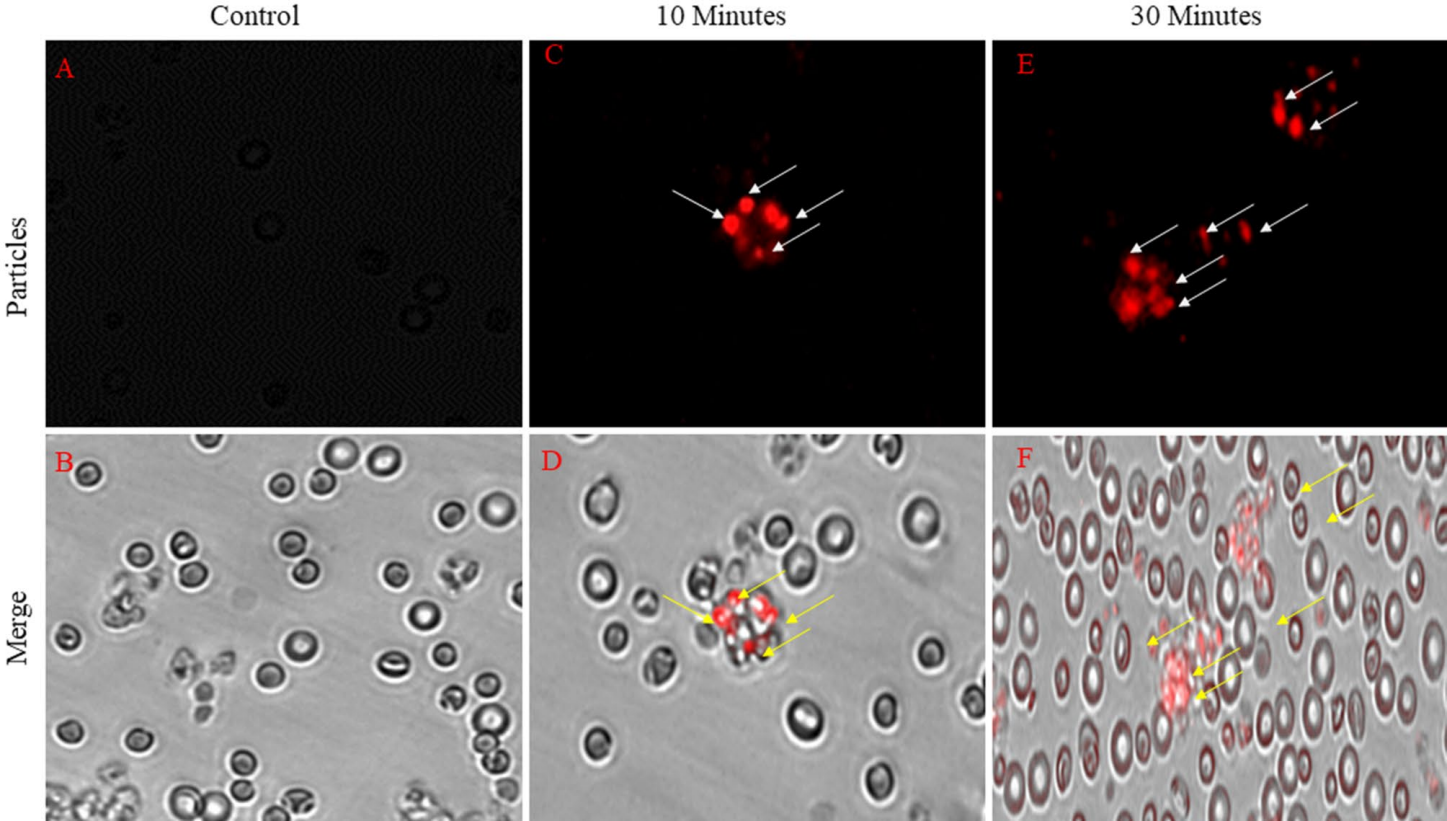
Evaluation of in-vivo Phagocytic uptake of the Rhodamine labelled Drug loaded Inhalable microparticles within Alveolar Macrophage cells. (**A**) Control cell, (**B**) 10 Min, (**C**) 30 Min.

We found that these particles have significant aerodynamic diameter as well as geometric standard deviation almost 1–5 μm of particles making them suitable for inhalation [Fig. 7]. In vitro phagocytic uptake of rhodamine-B labeled microparticles were seen to be readily phagocytosed by human macrophage at both time point at [Fig. 8]. For the further in-vivo inhalation it confirmed that particles are amenable to lungs alveolar macrophage cells in terms of size and shape. They have greater propensity to reach the lungs and get phagocytosed by alveolar macrophage cells. The isolated BAL fluid from mice lungs. It's clearly seen in phagocytosed cells with emission of red fluorescence which is indicated by white arrow in confirming that the particles are readily phagocytosed by alveolar macrophages in a time dependent manner [Fig. 9].

### In-vitro cytotoxicity

**Figure 10:**
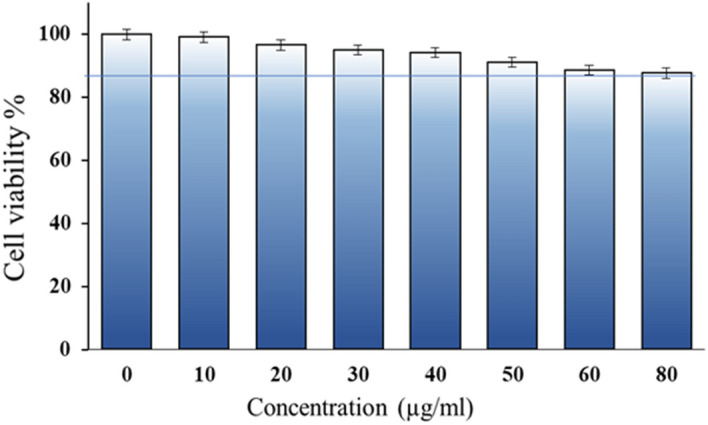
In-vitro cell viability assay within human macrophage cell.

The cell viability evaluation of drug loaded [DYDGP] did not show any cytotoxicity to THP-1 cells post 24 h exposure at 10 μg/ml concentration and percentage of the cell viability was found to be almost 90% upto a concentration of 50 μg/ml [Fig. 10]. Further slight decline in cell viability was seen at higher concentrations that were insignificant upto 80 μg/ml, indicating that the particles are fairly biocompatible in nature and thus safe for exposure to cells and tissues.

## Discussion

Conventional TB therapy requires administration of high dose of anti-TB drugs for prolonged duration, leading to non-compliance issues and the emergence of the multi-drug resistance. A large number of recent studies have shown that pulmonary drug delivery of particles in form of dry powder inhalations [DPI], allows delivering drugs directly to lungs, and results in local drug concentration much higher than that achievable by oral administration. As opposed to building up high drug concentrations in blood by conventional oral treatment, this facilitates targeting of anti-TB drugs to alveolar macrophage harboring mycobacteria, builds up high intracellular drug concentrations and thereby enhances drug efficacy^12^. Thus, a great deal of research has attempted to generate aerosols for inhalation and deposition to lungs, or design rational “smart” particles based on the biological information on barriers^23–25^. Glucan particles offer dual advantage as biocompatible and biodegradable immunomodulatory particles that boost the immune function during various immune challenges and also allow encapsulation of nano-sized molecules including drugs, proteins, DNA and RNA. Additionally, the glucan nature of these particles permits their receptor mediated endocytosis by macrophage. Our previous studies have shown that RB loaded glucan particles confer enhanced protection against *M.tb.* as compared to that by equivalent amount of free drug^12,34–37^. Considering the potential of inhalable particles to reach alveolar macrophage and deliver drugs, we attempted to develop an inhalable Rifabutin loaded, β-glucan particle-based drug delivery system for Pulmonary TB, the most common form of TB. Spray drying technology has been extensively used to prepare inhalable, uniform structured particles in a narrow size distribution. The preparation of RB loaded glucan particles by spray drying process generated by the cyclone, yielded uniform, hollow spherical particles that shriveled in shape after drying. The delivery of drug loaded particles to deep lungs mainly depends on the flow properties of the particles. Inter-particle agglomeration can hinder the alveolar penetration attributed to static charges, even though spray dried micronized particles have small aerodynamic diameters. The addition of amino acid leucine has been documented to enhance the flow property of particles by reducing inter-particle cohesion force and lowering the agglomeration issue of particles^20,38,39^. Additionally, conventional DPI additives such as leucine and lactose generally improve the properties of formulations such as uniformity and pulmonary penetration. The uniform, poly dispersed, and better yield of RB loaded glucan particles is therefore attributed to the addition of leucine during the preparation process. The presence of active excipients like leucine and alginate layer on the drug loaded particles is expected to enhance their thermal behavior as compared to the extracted and standard glucan particles^40^. Drug loaded alginate sealed β-glucan particles exhibited significant higher thermal stability as compared to other formulations due to presence of the triple helical structure of glucan particles which is assumed to provide better thermal strength^40^. Differential scanning calorimetry of the particles reveals the phase transition temperature above T_m_ are known to undergo oxidation and thermal degradation. The DSC data also showed that melting temperature [T_m_] of the standard and extracted glucan are almost the same. The DSC and FTIR data thereby confirmed that the extracted glucan particles do not have any impurity and biochemical discrepancy. The particles with smaller diameter size have a higher chance to reach deep in the lungs, extra fine particles are usually exhaled out during respiration. The optimum size range of the particles reported to readily reach the deep lung area lies in the range of 0.5–5.0 µm diameter^22^.The formulations have been found to have sufficient aerodynamic property as showed in mass deposition. According particle deposition on each stage of the cascade impactor, deposition profile of particle and MMAD data has shown particles appear to be suitable for deep lung delivery^40,41^. The size and shape of particles indicate that these are amenable for receptor mediated uptake by macrophage. Our in-vitro/in-vivo studies have shown these particles are fairly biocompatible in nature and are efficiently phagocytosed by both alveolar as well as human macrophage cell. Recent studies has indicated that rifabutin loaded inhalable microparticle have found the MMAD and GSD value under 5 μm and having the fine particle fraction (FPF) is almost 29–30%^8,42,43^.

Since optimizing aerosol deposition is important but insufficient for pulmonary delivery, multifunctional “smart” particles are desirable to meet the higher delivery requirements. However, there is still a long way before these particles be available in market. Deeper understanding of the obstructive lung diseases is needed for particle development. Moreover, there are several issues needed to be clarified before the designed particles entering into the clinical use, such as safety of excipients, potential toxicity of nanoparticles on the immune system and differences of lung barriers between animals and human. Comprehensive studies are need in these fields in the future.

RB loaded inhalable β-glucan particles hold great promise as a pulmonary drug delivery system for the treatment of tuberculosis and mycobacterial infections. By addressing the challenges associated with rifabutin delivery, such as poor solubility and low bioavailability, while leveraging the advantages of β-glucans, this innovative approach offers the potential for improved therapeutic outcomes, enhanced patient compliance, and reduced systemic side effects.

Further research and development efforts are warranted to optimize formulation parameters, evaluate efficacy in preclinical and clinical studies, and navigate the regulatory pathway toward clinical translation and commercialization. Thus, this formulation holds a promising prospective for carrying large doses of drug, providing targeted delivery with the minimal side effects. Our further interest to evaluate the intra cellular immune response mediated by these particles within *mycobacterium* infected host macrophage cells to target residing mycobacteria.

## Methods

All methods were performed in accordance with the relevant guidelines and regulations.

### Ethics statement

Animal experimentation performs in this study as per ARRIVE guidelines (https://arriveguidelines.org). Institutional Animal ethical committee approval obtained from Chhatrapati Shahu Ji Maharaj University, Kanpur [IAEC approval no. IAEC/UIP/August2023/025 dated 12.08.2023]. For experimentation we used healthy Balb/c mice and maintained in institutional animal house under 12 h dark/light condition along with bottle of water and feeding pellet in animal cage with optimum parameter. For the isolation of broncheo-alveolar lavage [BAL Fluid] mice euthanized and disposed as per biomedical waste management facility, Chhatrapati Shahu Ji Maharaj University, Kanpur.

### Reagents and solutions

For the extraction of β-glucan, baker’s yeast was purchased from a local shop in Lucknow. Anti TB drug, rifabutin was procured from Lupin pharmaceuticals, and other chemicals such as l-leucine, acetone, isopropyl alcohol, HCl [analytical grade], DMSO were purchased from Merck. All cell culture reagents including FBS, RPMI-1640, antibiotic–antimycotic mixtures were purchased from Gibco. PMA was purchased from thermo fisher scientific, rhodamine and DAPI from sigma. All aqueous solutions were prepared in triple distilled water [TDW].

### Cell culture

For in vitro study human macrophage cells [THP-1] was procured from the National Centre for Cell Science [NCCS], Pune and cells were cultured within RPMI-164 media supplemented with 10% FBS and 1% antibiotic and antimycotic solution. For differentiation of macrophage cell incubated with phorbol myristic acid (PMA) for 48 h after that cell are ready for further experimentation. All the working reagents were prepared in milli-q water and working stocks were stored as per manufacturer instructions at − 20 °C for further processing.

### Preparation of L-leucine added Rifabutin loaded inhalable β-glucan microparticles

Glucan microparticles were prepared by slight modification of the alkaline acidic extraction [AAE] method as described previously^12^. Briefly, 20 g of baker’s yeast was suspended and 1 M NaOH, heated at 60 °C, for 1 h followed by centrifugation at 200 × *g* for 10 min at room temperature. Then the sediment was treated with acidic water for the removal of mannosylated proteins and subsequently washed 4 time with isopropyl alcohol and 2 time with acetone and centrifuge for 10 min. After that semisolid mixture was sonicated for 1–2 min to avoid the formation of microparticulate agglomeration. Now we add the l leucine for the improvement of flow property of microparticle and also to overcome the aggregation by lowering adhesive forces within microparticles. Now sediment material were spray dried by BUCHI 290 Mini spray dryer Switzerland) under stirring conditioned. The spray drying parameter inlet/outlet temperature, aspirator setting and feed rate were used as per described in [Table 1], to get obtain white colored blank β-glucan microparticles [YDGP].

For the drug loading rifabutin [RB] drug stock were prepared in 0.1N HCl solution and incubate at room temperature for 1 h then after added tris buffer and centrifuge at 10000 rpm for 10 min Usually glucan microparticle was hollow and pours in nature so for the retention of entrapped drug pores sealing was done by 0.25% sodium alginate and CaCl_2_ solution was added drop wise manner under stirring conditioned after that spray dried and we get obtain drug loaded alginate sealed violet coloured microparticles [DYDGP] [Fig. 1].

### Fluorescence tagging

For in vitro/in-vivo phagocytic uptake and inhalation assay fluorescence tagging of these microparticles was done by rhodamine-B red dye. Initially 10 mg/ml rhodamine stock was prepared in distilled water then added particle and allows incubating at room temperature in dark conditioned. After that centrifuge and washed excess dye to removed form particle and final fluorescence microparticles [YDGPRD] was stored in the 4° for further experimentation use^12^.

### Biophysical characterization

#### Particle size

Particle size was determined by Laser scattering [Malvern Mastersizer] from IIT, Kanpur as previously described in^12,18^. Briefly, blank and drug-loaded particles mixed with an equal amount of sodium dodecyl sulfate [SDS] and suspended in 1 ml milli-Q water, were subjected to laser obscuration at a factor of > 10% for size determination by laser scattering.

#### Scanning *electron* microscopy

For the morphological analysis of these particles were carried out from Electron microscopy unit, SAIF facility at CSIR-CDRI, Lucknow. Microparticle was visualized by scanning electron microscope [FEI Quanta 250] at an accelerated voltage of 15 kV at various magnifications.

#### Fourier transform infra-red [FTIR] spectroscopy

Functional group analysis of the prepared microparticles was carried out by FT-IR spectrometer [Perkin Elmer]. For both blank and drug loaded particles were compressed with KBr at a ratio of 1: 15 (w/w, sample; KBr) and FTIR spectrum scanning range from 400 to 4000 cm^−1^ were recorded.

#### Differential scanning calorimetry [DSC]

The thermal stability of yeast, standard β-glucan, extracted blank β-glucan, pure rifabutin, and drug loaded particles was determined by differential scanning calorimetry. For this [5 mg sample] were placed in an aluminum pan [TA instruments discovery model DSC-25, new castle, DE.USA], which was then hermetically sealed and allowed the determination of glass transition T_g_, melting point T_m,_ and enthalpy change ΔH_m_. The empty pan were used as a reference in the furnace, heating range of sample pan was from 30 to 200 °C and flow rate was kept at 5 °C min^−1^, when required, using an nitrogen with flow rate of 10 cm^1^ min^**-**1^.

#### Quantification of drug

Quantification of incorporated drugs within microparticles was carried out by HPLC (Shimadzu (Japan) Class VP HPLC system with a Luna C18 column (5 m, 4.6 mm × 250 mm, Phenomenex, Torrance, USA with slight modification of the protocol as reported by^12,19^. Briefly, we prepared the mobile phase by mixing acetonitrile: methanol [45:55], which was filtered through a 0.22 µm filter and pump flow rate was kept at 1 ml/min RB was seen to be eluted at 8.5 min by monitor by using the UV detector set on 275 nm. The standard curves were generated in the concentration range of 2–12 µg. Rifabutin was extracted from the drug-loaded-alginate sealed YDGP suspended in 0.01 N HCl, and then diluted in mobile phase after filtration through a 0.22 µm filter. The drug loading efficiency [LE] for RB within the microparticles was calculated as follows:

$$\text{Drug loading efficiancy }[\%]=\frac{\text{Amount of drug in formulation}}{\text{Expected drug}}\times 100$$

#### Evaluation of aerodynamic behaviour of particles

Aerodynamic behaviour of the microparticles was carried out from the Inhalation Toxicology Facility, Indian Institute of Toxicology Research [CSIR-IITR], Lucknow. Flow property of particles was evaluated through MOUDI 100 NR cascade impactor (TSI Incorporated, USA). Particle size distribution analysis was performed using 10 stage (S1–S10; cut off size of 18 µm, 10 µm, 5.6 µm, 3.2 µm, 1.8 µm, 1.0 µm, 0.56 µm, 0.32 µm, 0.18 µm, 0.10 µm, and 0.056 µm) at 28 L/minute using MOUDI cascade impactor attached with handheld inhalable dry powder disperser device (which was used to administer microparticles in animals). The analysis was performed in triplicates using 10 mg of blank [YDGP], and drug loaded [DYDGP] for single analysis for 15 s. The total mass retained at each stage cut off size was calculated by taking the difference between post and pre weight of aluminium foil used at each stage (S1–S10). A log graph (Fig. 6b) was plotted for percent (%) cumulative mass and cut off size of each stage and the particle size at which the line crosses the 50% mark was taken as estimated mass median aerodynamic diameter (MMAD). And the particle size at which the line crosses the 84.1% mark and the 50% mark was noted. For the calculations of geometric standard deviation (GSD) refer to the above log probability graph GSD was calculated using formula as follows:

$$\text{GSD}=\frac{84.1{\% \text{mark} }}{50{\% \text{mark}}}$$

#### In-vivo phagocytic uptake of the particles

To investigate these microparticles are inhalable or not, we performed in-vivo inhalation of these microparticles in 4-week-old, 6–8 g BALB/c mice using the inhalation apparatus as described previously^19,44–46^. Briefly, 10 mg of fluorescent tagged GP-RD particles were kept in inhalation apparatus for the nasal inhalation to mice for following time points 10–30 min. after that euthanize mouse by isoflourane immediately dampen animal fur with 70% ethanol. By using scissors make a small incision in the animal skin at the neck, open toward the sides and upwards toward the mouth. Peal skin upwards to expose the neck. Cannulate the trachea using a surflo catheter. Keep one hand on the catheter while proceeding with the lavages. Alternatively, stabilize trachea with forceps throughout the procedure or tie the trachea around the catheter using sutures. Load a 1 mL syringe with 0.5 mL sterile ice-cold Mg^++^ and Ca^++^ free PBS-EDTA. Place syringe in the end of the surflo catheter and carefully inject saline into the animal lungs. Aspirate saline by pulling barrel of syringe. Remove syringe from the catheter, eject recovered lavage fluid into 15 mL centrifuge tube kept on ice. Centrifuge remaining lavage at 450 g for 10 min and discard supernatant. Obtain alveolar macrophage cells that were dispensed in 6 well plates for visualization of microparticle uptake under fluorescence microscope at 100x.

#### In-vitro phagocytic uptake of the particles

In vitro phagocytic uptake of the microparticles was analyzed using human macrophage THP-1 cells. 1 × 10^4^ cells were seeded in each 96 well plates and adhered by using [50 ng/ml] PMA and plates were incubated for 48 h for proper cell adherence. After that PMA containing media was discarded and washed with incomplete RPMI-1640 media and incubated for 24 h with complete media. Subsequently, the cells were incubated with fluorescent tagged particles for 10–30 min for microparticle uptake. After that cell were washed thrice with 1X PBS and visualized under Fluorescence microscope at 100X.

#### In-vitro cell viability assay

The viability of THP-1 cells post-exposure to the aforementioned formulation was evaluated by MTT assay. Briefly, 1 × 10^4^ cells were seeded in each 96 well plate and adhered by [50 ng/ml] Phorbol Myristic Acid [PMA]. Cells were treated with different concentration of particles [10–80 μg/ml] and plates incubated overnight. Thereafter, 100 μl MTT dye was added for 3–4 h, after that 100 μl DMSO was added to dissolve the formazan and plates were read at 570 nm using ELISA Plate reader. Cell viability was calculated using the following formula:

$$\text{Cell viability }[\%]=\frac{\text{Absorbance of sample }}{\text{Absorbance of control}}\times 100$$

### Statistical analysis

Statistical analysis was done by GraphPad prism software by applying the One-way analysis of variance [ANOVA]. All data has been taken in a triplicate and expressed as mean ± standard deviation. The data was considered to be significant if *p* < 0.05.

## Data Availability

The datasets used and/or analyzed during the current study available from the corresponding author on reasonable request.
